# Supplementary figures and images for: Analysis of metabolomics and transcriptomics data to assess interactions in microalgal co-culture of Skeletonema marinoi and Prymnesium parvum
Source: PLoS One. 2025 Jul 28;20(7):e0329115. doi: 10.1371/journal.pone.0329115 (PMC12303313; doi:10.1371/journal.pone.0329115)

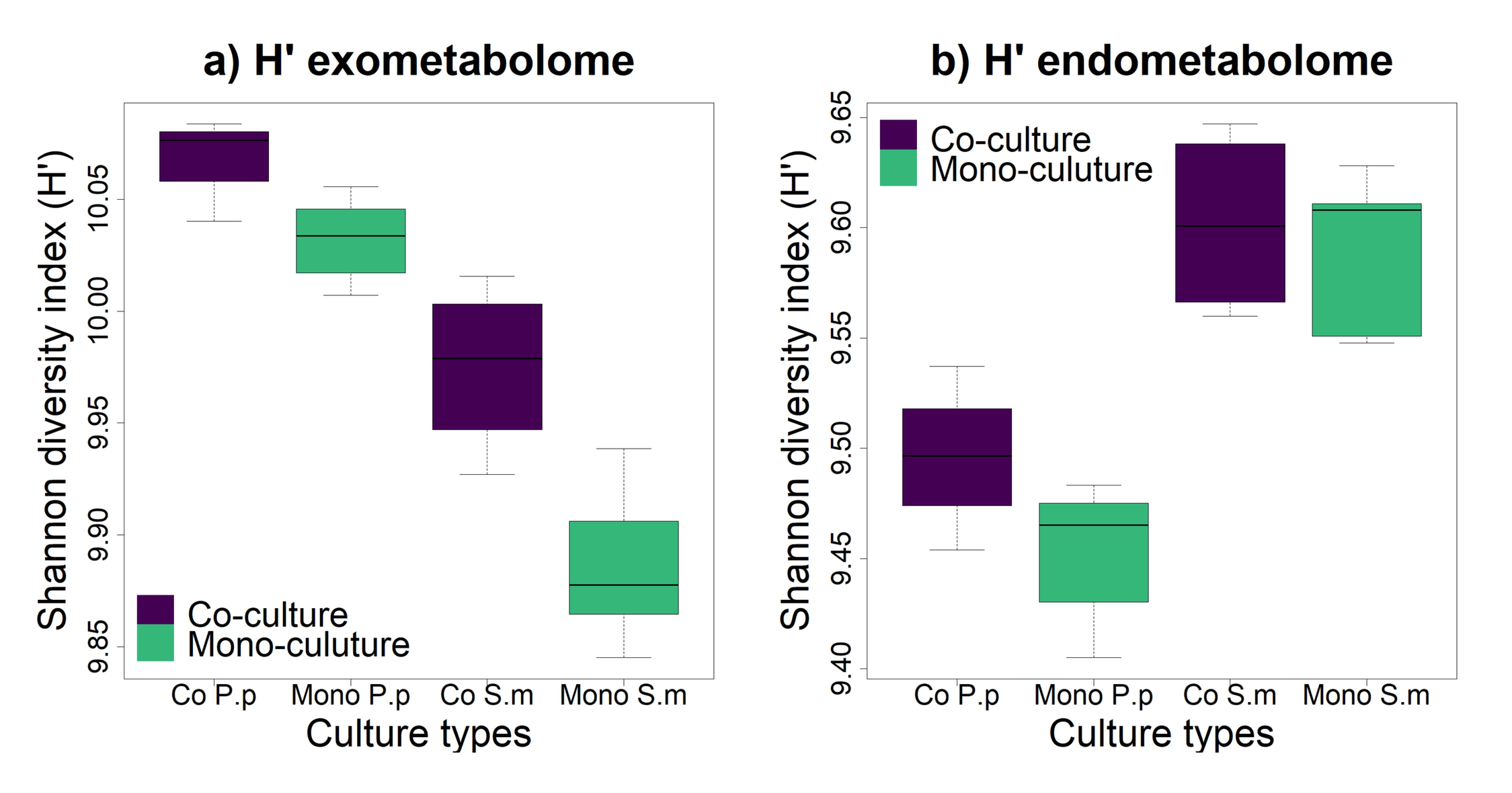

Supplement: S1 Fig — Co-culture samples are coloured purple and mono-culture samples are coloured green. a) exometabolome feature diversity with the left two samples showing P. parvum and the right samples S. marinoi. b) endometabolome feature diversity with P. parvum on the left and S. marinoi on the right. Mono = mono-culture, Co = co-culture, P.p = P. parvum, S.m = S. marinoi. (TIF) [file pone.0329115.s003.tif]

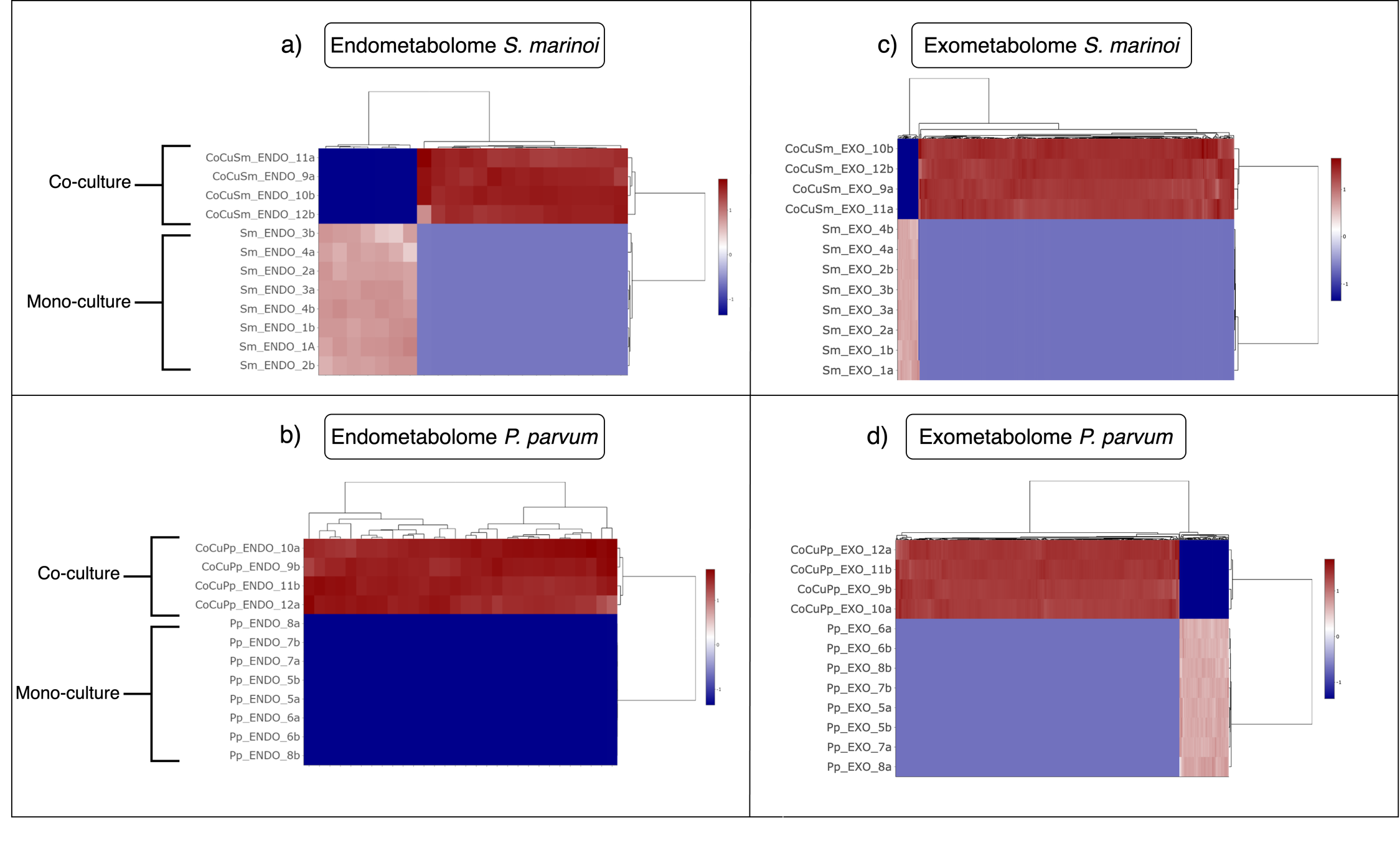

Supplement: S2 Fig — The dendrogram clusters the conditions and features based on their intensities, with red indicating high intensity, white indicating no change, and blue representing low intensity or absence. a) For endometabolome, 22 differentially abundant features were selected for S. marinoi, out of which only seven were abundant in mono-culture. b) Conversely, all 30 differentially abundant features detected for P. parvum endometabolome were abundant in co-culture conditions. For exometabolome, a higher number of differentially abundant features were detected for both species, with more abundant features found in co-culture of S. marinoi and P. parvum. c) In total, the S. marinoi differentially abundant features were 324, out of which 304 features were abundant in co-culture. d) For P. parvum, the total number was 491, out of which 418 features were abundant in co-culture. (TIF) [file pone.0329115.s004.tif]

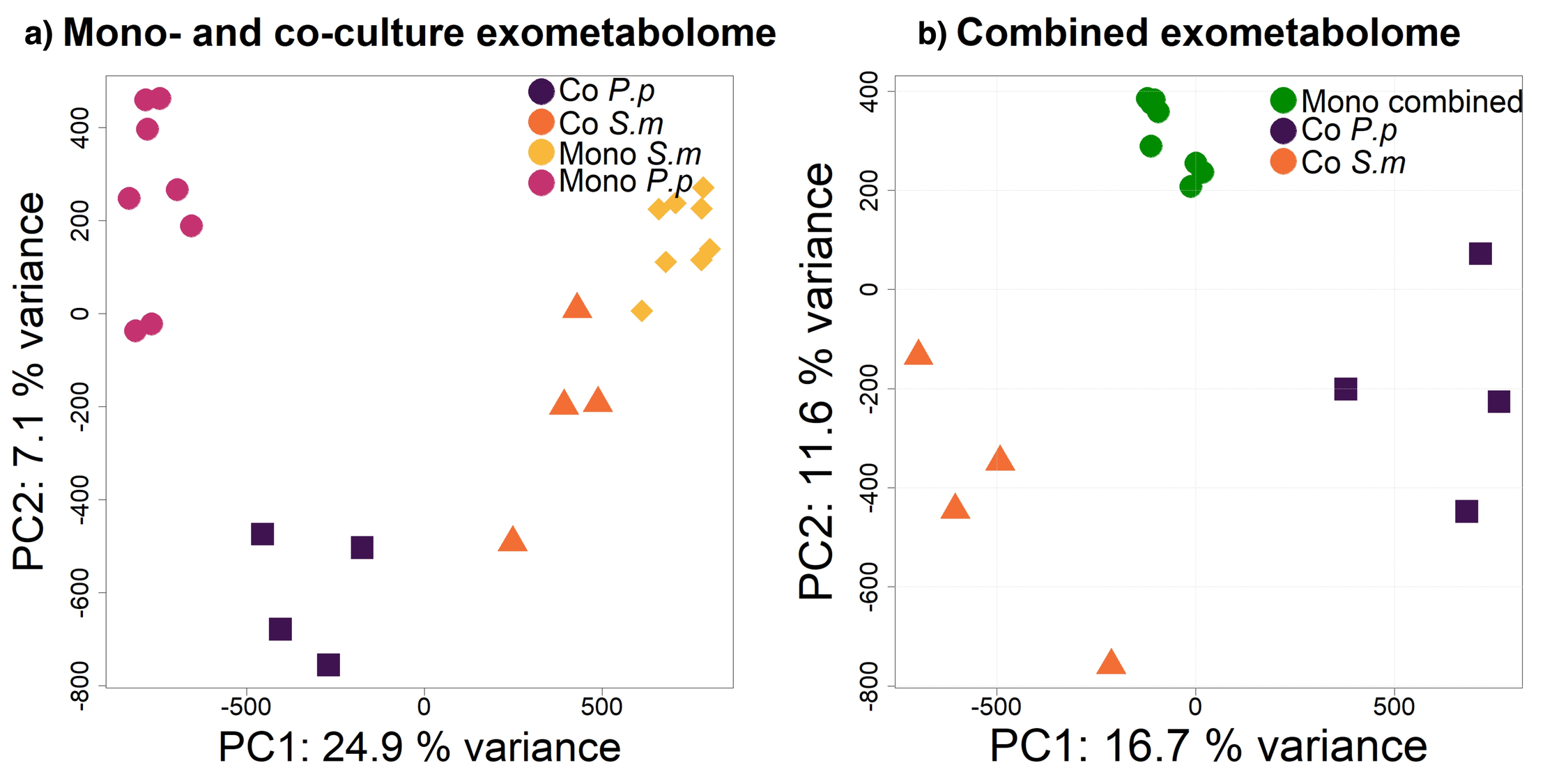

Supplement: S3 Fig — a) Shows co-culture (orange for S. marinoi and purple for P. parvum) and mono-culture (yellow for S. marinoi and pink for P. parvum) samples with principal component (PC) 2, explaining 7.1% of the variance, plotted against PC2, explaining 24.9% of the variance. b) Shows combined mono-culture for the S. marinoi and P. parvum exometabolome in green, while the S. marinoi co-culture is shown in orange, and P. parvum co-culture is shown in purple, where PC 2 describes a variance of 11.6%, and PC1 describes a variance of 16.7%. The PCA in b) clearly distinguishes between the combined mono-cultures and individual co-cultures, indicating metabolic transformation occurring in the co-culture of both species. (TIF) [file pone.0329115.s005.tif]
